# On the Consequences of Reporting from Medical Societies

**Published:** 1829-04-01

**Authors:** 


### Reporting from Medical Societies.

If we merely regard the sources whence these reports spring, the subject is of very local interest, there not being more than three or four medical societies in this metropolis where Reporting is carried on;—but if we look to the wide circle of the profession over which these reports circulate, the importance of the subject will be readily appreciated. Our readers are aware that we have always advocated the propriety and the advantage of reporting the *oral discussions* of medical societies—and even the substance of papers not intended for publication in the transactions of societies. We are still of the same opinion, as far as fair and discreet reporting is concerned; but every day's experience convinces us of the great inconveniences, not to say fatal consequences of such reports, if not guarded by some salutary checks.

In the following brief observations we declare that we have no particular society—and much less any particular individuals in view;—but yet we apprehend that there are few medical societies in this metropolis, where the truth of our remarks will not be felt. The object of a medical society is to draw together a number of medical practitioners, where papers may be read, cases related, and these papers and cases discussed. It never could have been originally contemplated that these oral discussions should go abroad in the form of printed reports, even among professional men, and still less among the non-professional world. Before this last step was taken, the members of societies met for the purpose of conveying and receiving mutual information in the form of colloquial and confidential communications. Under such a system, restraint was taken off, and a generous confidence was imposed mutually in each other, that professional discourses should not pass beyond the walls of the society. Such a system led naturally to a relation equally of successful and *unsuccessful* cases—and it offered no temptation to Puffery, the bane and disgrace of the medical profession! The system of Medical Reporting has, under existing circumstances, effected a complete revolution in the ordinary affairs of medical societies—or, at least, it is rapidly tending to such revolution. Instead of the experienced, the talented, and the candid practitioners who formerly disclosed the results of their observations in such societies, we have now a premium held out for the Publication of wonderful cures and cases, by men who are aiming at notoriety, and whose mottoes are consequently—

"Non mundo sed sibi."

Formerly when a paper was read, or a case detailed by any member, a scientific discussion arose on the merits of the case, the nature of the disease, the utility of the remedies and so forth. What do we now see? The moment the paper or the case is heard to an end—and that with evident impatience—a host of practitioners are ready to start on their legs—not to discuss the paper or case detailed, but at once to launch forth into the relation of cases of their own, which they modestly preface as bearing on, or elucidating that before the society, but which in general, bears as much analogy to the subject under discussion as a gas-lamp does to a hackney-coach! The causes of this strange perversion of order and design, are as clear as the sun at noon-day. Upon the present constitution of things, a portion of medical society resort to these meetings for the sole purpose of appearing in print on the succeeding Saturday. Every moment spent in listening to their brethren, or in discussing subjects where they might perchance exhibit deficiency, is so much time lost—and the grand manœuvre is to out-general their neighbours, and crowd as many cases on the minutes of the REPORTER's book as the time of the society will permit! The consequences of such a system may be very easily appreciated. If some salutary check be not put upon it, the societies in which it is encouraged or even permitted will stand a fair chance of being ruined. This will be the case to a greater or less extent under the most impartial system of reporting—because the system itself holds out the temptation to puffery. But where a partial and venal press, influenced solely by prejudice or pelf, lends its aid, by distorting and ridiculing the statements of one party, and glossing over, or dressing up the egotisms of another, the injurious consequences will be greatly aggravated, and they will operate, not merely by driving away or deterring the most useful members of medical societies, but, by poisoning the springs of medical science, they will carry error and deception through every ramification of the profession.

The PRESIDENTS of these associations have an arduous task to perform, in restraining the interested egotism, the vain lucubrations, and the irregular ramblings of the members. One cardinal point which they should always keep in view, is to check the DIGRESSIVE NARRATIVES, until all members who wish to discuss the *principles* or the practical points of the question, have had an opportunity of speaking. This discussion of principles and practice gives dignity to a meeting of medical men, and presents a very effectual barrier to the selfish views of the egotists. Neither should *irrelevant* cases be permitted to be *smuggled* in by these candidates for hebdomadal fame, till the discussion of the subject before the society is fairly exhausted. It is then quite soon enough to allow the irregular, desultory, and promiscuous statement of cases, to eke out the time which cannot be better employed in the more elevated pursuits of scientific associations.

We have thrown out the above hints, of a very general nature, and we sincerely hope they may have a beneficial effect in the quarters where they are designed to operate. As independent and disinterested spectators, however, we shall certainly take opportunities of making more special applications of criticism, should the evil increase, or even persist in its present course. But we trust this censorship may not be necessary—and that the good sense and proper feeling of members of a liberal profession will keep them from deviating into paths beneath the dignity of science.

